# Does Point-of-Care Ultrasound Affect Fluid Resuscitation Volume in Patients with Septic Shock: A Retrospective Review

**DOI:** 10.1155/2024/5675066

**Published:** 2024-05-06

**Authors:** Enyo A. Ablordeppey, Amy Zhao, Jeffery Ruggeri, Ahmad Hassan, Laura Wallace, Mansi Agarwal, Sean P. Stickles, Christopher Holthaus, Daniel Theodoro

**Affiliations:** ^1^Department of Anaesthesiology, Washington University School of Medicine, St. Louis, MO, USA; ^2^Department of Emergency Medicine, Washington University School of Medicine, St. Louis, MO, USA; ^3^Washington University School of Medicine, St. Louis, MO, USA; ^4^Division of Biostatistics, Washington University School of Medicine, St. Louis, MO, USA

## Abstract

**Background:**

Fixed, large volume resuscitation with intravenous fluids (IVFs) in septic shock can cause inadvertent hypervolemia, increased medical interventions, and death when unguided by point-of-care ultrasound (POCUS). The primary study objective was to evaluate whether total IVF volume differs for emergency department (ED) septic shock patients receiving POCUS versus no POCUS.

**Methods:**

We conducted a retrospective observational cohort study from 7/1/2018 to 8/31/2021 of atraumatic adult ED patients with septic shock. We agreed upon *a priori* variables and defined septic shock as lactate ≥4 and hypotension (SBP <90 or MAP <65). A sample size of 300 patients would provide 85% power to detect an IVF difference of 500 milliliters between POCUS and non-POCUS cohorts. Data are reported as frequencies, median (IQR), and associations from bivariate logistic models.

**Results:**

304 patients met criteria and 26% (78/304) underwent POCUS. Cardiac POCUS demonstrated reduced ejection fraction in 15.4% of patients. Lung ultrasound showed normal findings in 53% of patients. The POCUS vs. non-POCUS cohorts had statistically significant differences for the following variables: higher median lactate (6.7 [IQR 5.2–8.7] vs. 5.6], *p* = 0.003), lower systolic blood pressure (77.5 [IQR 61–86] vs. 85.0, *p* < 0.001), more vasopressor use (51% vs. 34%, *p* = 0.006), and more positive pressure ventilation (38% vs. 24%, *p* = 0.017). However, there were no statistically significant differences between POCUS and non-POCUS cohorts in total IVF volume ml/kg (33.02 vs. 32.1, *p* = 0.47), new oxygen requirement (68% vs. 59%, *p* = 0.16), ED death (3% vs. 4%, *p* = 0.15), or hospital death (31% vs. 27%, *p* = 0.48). There were similar distributions of lactate, total fluids, and vasopressors in patients with CHF and severe renal failure.

**Conclusions:**

Among ED patients with septic shock, POCUS was more likely to be used in sicker patients. Patients who had POCUS were given similar volume of crystalloids although these patients were more critically ill. There were no differences in new oxygen requirement or mortality in the POCUS group compared to the non-POCUS group.

## 1. Introduction

Patients diagnosed with septic shock in the emergency department (ED) carry a high risk for morbidity and mortality. A dysregulated host response to infection that results in new or worsening organ dysfunction is sepsis by definition [1, 2] and carries mortality rates that can reach 20% or higher in the inpatient setting [1, 3]. Risk-adjusted mortality continues to vary greatly between regions and hospitals despite many national and systemic initiatives, indicating there may be opportunities to optimize and improve care [1]. Early recognition with appropriate hemodynamic management and source control is regarded as imperative in the management of sepsis [1.4]. The 2021 Surviving Sepsis Campaign guidelines recommend early, fixed, large volume (30 ml/kg) intravenous fluids (IVFs) administration in cases of suspected severe sepsis or septic shock within the first 3 hours of clinical presentation [4, 5]. However, while ample fluid resuscitation is a crucial part of early goal-directed therapy, adoption of a fixed volume regimen across all patients risks over-resuscitation and fluid overload, especially in patients with congestive heart failure (CHF) and end-stage renal failure (ESRD) [6–8]. Adverse effects of over-resuscitation include hypervolemia and increased oxygen requirement, potentially resulting in further medical interventions or even death [9]. These risks call for effective monitoring and assessment of volume with hemodynamic status changes, which is difficult based on clinical and laboratory evaluation alone [10–12].

Point-of-care ultrasound (POCUS) including echocardiography and lung ultrasound has been used to assess volume and guide hemodynamic management with IVF administration [13–15]. Volume overload can present as increased extravascular lung water, which can be accurately detected via POCUS prior to clinical symptoms [16–18]. A number of studies have shown a correlation between increased extravascular lung water and mortality in the critically ill [19, 20]. As such, POCUS of the lung has been suggested as an additional tool that can rapidly and accurately identify early increased extravascular lung water (pulmonary edema) that develops when patients are over-resuscitated, providing a signal in the risk-benefit consideration for further volume expansion [21, 22].

The primary study objective was to evaluate whether the total IVF volume given differs for septic shock patients receiving POCUS versus no POCUS in the ED. Secondary objectives included 'evaluation of new oxygen requirements, overall mortality, and outcomes in a subgroup of patients with CHF and severe renal failure.

## 2. Methods

This study was performed according to the Strengthening the Reporting of Observational Studies in Epidemiology (STROBE) guidelines for observational studies (Supplemental File 1) [23]. The Institutional Review Board reviewed and approved the study.

### 2.1. Setting and Subjects

We conducted a retrospective cohort study of patients who presented to the ED between 7/1/2018 and 8/31/2021 according to standards of chart review in case selection, abstractor training, monitoring and blinding, and interrater agreement to improve accuracy and minimize inconsistencies [24, 25]. The setting was a large (∼1200 hospital beds) urban academic, residency-affiliated, tertiary care medical center. Patients were included in our analysis if they were 18 years old or older, with a diagnosis of sepsis. Sepsis was defined using the Systematized Nomenclature of Medicine-Clinical Terms (SNOMED-CT) [26, 27], an internationally maintained hierarchical terminology system. The label was set to 1 if the parent concept for sepsis, SNOMED-CT identifier (SCTID = 91302008), or any of its descendants was found in the problem list. To increase the likelihood that patients were critically ill and being treated for severe sepsis or septic shock, we restricted inclusion to patients who had both Centers for Medicare and Medicaid Services (CMSs) Severe Sepsis/Septic Shock (SEP-1) fluid requirement criteria of 30 cc/kg fluid bolus within 3 hours of presentation: a lactate ≥4 and hypotension (SBP <90 or MAP <65) [28, 29]. POCUS was performed at the discretion of the bedside clinical team and was included in the analysis if a POCUS report was available in the medical chart. In this study, POCUS is performed by the emergency medicine (EM) team, which can include EM residents, ultrasound fellows, and or EM faculty. Furthermore, if POCUS is performed by other trainees during their clinical role in the ED, each ultrasound image is reviewed with an EM faculty who are all proficient in basic cardiac and lung POCUS. In addition, advanced EM ultrasound faculty provides weekly ultrasound image quality assurance for each ultrasound study performed in the ED. Using standardized abstracted forms, trained chart abstractors reviewed electronic medical records for comorbidities (CHF, hypertension, diabetes, chronic obstructive pulmonary disease, severe renal failure defined as renal failure greater than stage 3 chronic kidney disease, and coronary artery disease); disposition and mortality; ED vital signs; total IV fluids received; vasopressor initiation; new oxygen requirement; and reported POCUS results.

### 2.2. Data Collection

Chart abstractors were trained using a standardized abstractor form to guide data collection and minimize bias including explicit criteria for case selection or exclusion. The performance of the chart abstractors was monitored by the lead study investigator (EA). Variables were defined a priori to minimize ambiguity, and clarifications and inconsistencies were discussed until consensus was reached among team members (EA, CH, and DT). Ten-percent random samples of abstracted data were reviewed for agreement and reliability.

### 2.3. Outcomes

The primary outcome was total IVF volume delivered to ED septic shock patients who had POCUS versus no POCUS. Secondary objectives included new oxygen requirements, overall mortality, and if these variables change in the subgroup of patients with CHF and severe renal failure.

### 2.4. Statistical Analyses

Continuous variables were expressed as medians with interquartile ranges (IQRs). Categorical variables were expressed as proportions and frequencies. A power analysis suggested that a minimum sample size of 300 patients would be required to detect an IVF difference of 500 milliliters between POCUS and non-POCUS cohorts, with a significance level of 5% to achieve power of 80%. The analysis also needed a 4 : 1 non-POCUS to POCUS ratio based on observed practice patterns in our ED. The Fisher exact test was used to compare differences between proportions. Bivariate comparisons were performed using chi-square or *t*-tests. A 2-tailed significance level of 0.05 was regarded statistically significant. All data were stored on a spreadsheet (Excel 2011; Microsoft Corporation, Redmond, WA), and analyses were performed with a commercially available statistical package (SPSS version 24; IBM Corporation, Armonk, NY).

## 3. Results

### 3.1. Patient Characteristics

**Figure 1:**
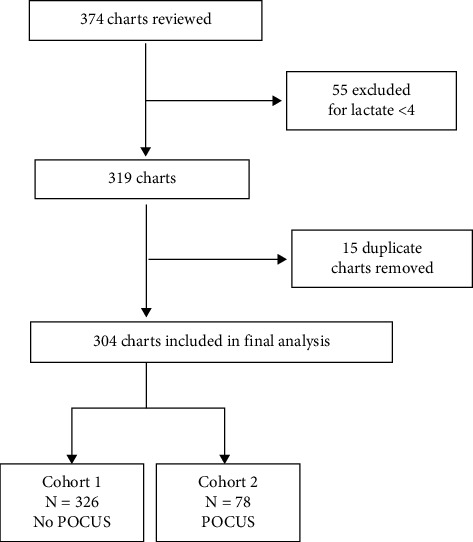
Chart review flow sheet. POCUS, point-of-care ultrasound.

**Figure 2:**
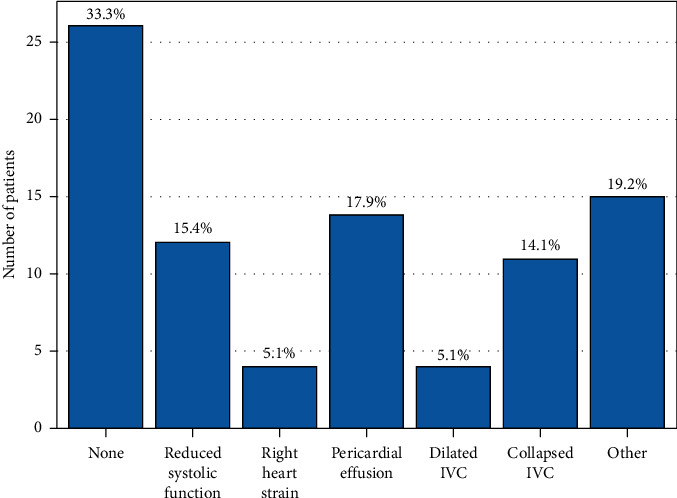
Cardiac POCUS findings in those with cardiac US, *n*=78^*∗*^ POCUS findings. POCUS, point-of-care ultrasound; US, ultrasound; IVC, inferior vena cava; ^*∗*^*n*=78 (61/78 had only cardiac ultrasound); IVC, inferior vena cava.

**Figure 3:**
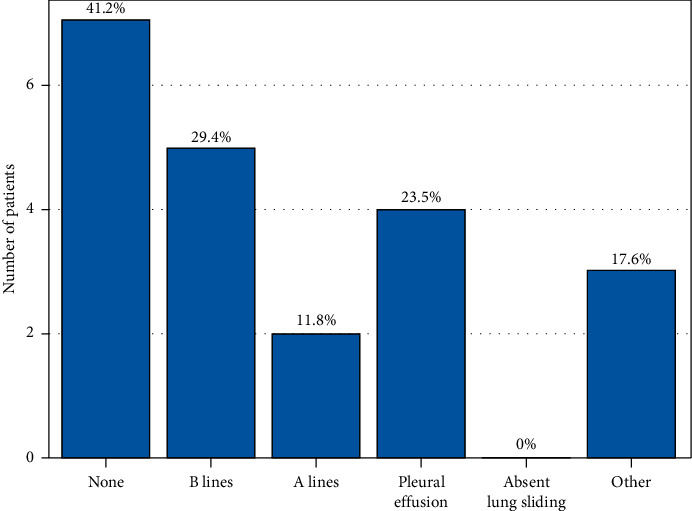
Lung POCUS findings in those with lung US, *n*=17^*∗*^; ^*∗*^*n*=17 (these patients all had cardiac ultrasound also).

We identified a total of 374 charts for review and 304 were included in the final analysis (Figure 1). There was 90% agreement in the random sample between the abstractors. This study's patient characteristics are presented in Table 1. Patients were predominantly male (56%), with a median age of 65 years. There were no statistical differences between POCUS and non-POCUS cohorts for age, race, gender, or comorbidities. History of CHF and/or severe renal disease was present in 42% of the POCUS group and 29% of the non-POCUS group. Figures 2 and 3 show the POCUS exam type and findings. Cardiopulmonary POCUS was used in 26% (78/304). Cardiac POCUS showed 15.4% (12/78) with reduced ejection fraction, 14.1% (11/78) with collapsed inferior vena cava (IVC), and 5.1% (4/78) with right heart strain. Lung ultrasound was used in 17/78 POCUS cases with 53% normal findings (none and A lines).

Primary and secondary outcomes are listed in Table 2. The POCUS vs. non-POCUS cohorts had statistically significant differences for the following variables: higher median lactate (6.7 [IQR 5.2–8.7] vs. 5.6 [IQR 4.7–7.4], *p*=0.003), lower systolic blood pressure (77.5 (IQR 61–86] vs. 85.0 (IQR 73–95), *p* < 0.001), more vasopressor use (51% vs. 34%, *p* = 0.006), and more positive pressure ventilation (38% vs. 24%, *p*=0.017). There was no statistical significance in patient outcomes between POCUS and non-POCUS cohorts including total IVF volume received in ml/kg (33.02 vs. 32.1, *p*=0.47), new oxygen requirement (68% [53/78) vs. 59% [133/226], *p*=0.16), ED death (3% vs. 4%, *p*=0.15), or hospital death (31% vs. 27%, *p*=0.48). History of CHF and/or severe renal disease was present in 42% of the POCUS group and 29% of the non-POCUS group. Similar distributions of lactate, total fluids, and vasopressors were found in CHF and severe renal disease subsets (Table 3).

Table 4 shows statistically significant predictors of POCUS in ED by univariable logistic regression, including the lactate level, systolic blood pressure (SBP), diastolic blood pressure (DBP), positive pressure ventilation, and vasopressors in ED with odds ratios of 1.1, 0.98, 0.98, 1.9, and 2.01, respectively. Furthermore, we performed stepwise logistic regression with all the covariates (except outcomes) to evaluate independent effects of the variables. The criteria used were a *p* value of 0.1 required for entry into the model and a *p* value <0.05 to stay. In Table 5, only lactate (OR 1.09, 95%CI 1.01–1.17) and SBP (OR 0.98, 95%CI 0.97–0.99) were consistently and strongly associated with POCUS use (data not shown).

## 4. Discussion

Our study demonstrates that septic shock patients with increased severity of critically illness were more likely to get POCUS during their ED resuscitation. However, both POCUS and non-POCUS groups received the same total fluid volume, and there was no difference between groups with regards to new oxygen requirement or mortality, even in subgroups with CHF and severe renal disease. This confirms that although POCUS utilization may be embraced, without clear protocols of care, the effect of POCUS on important patient-centric outcomes may not be immediately perceived and healthcare workers may be reluctant to depart from Surviving Sepsis Campaign recommendations.

First, our data confirm the selection bias that those patients receiving POCUS evaluations were physiologically more critically ill (higher lactate, lower blood pressure, more mechanical ventilation requirement, and vasopressor use in the ED). As has been demonstrated in prior randomized controlled and observational trials that lack clear POCUS-based protocols for care, it would be expected that those with baseline characteristics suggestive of more severe illness will receive more interventions, including fluids and POCUS [30–32]. Wang et al. used POCUS findings in a protocolized fashion in postresuscitated critically ill patients and found improved fluid balance and reduced ICU length of stay [33]. Unfortunately, in our study, the timing of these interventions in relation to POCUS was unknown (e.g., whether vasopressors were started before or after POCUS), and the retrospective nature of the study renders it difficult to draw further conclusions of how POCUS guided clinical management.

Second, our data showed that the total amount of fluids administered between the POCUS and non-POCUS groups in the acute resuscitation phase was the same even though sicker patients were more likely to receive POCUS. While there appears to be a lack of studies that specifically quantify volume resuscitation in the setting of POCUS use, there exists some literature with mixed findings on this topic. In a proof-of-concept study by Le Bastard et al., patients with sepsis who received POCUS to assess volume status received less than the recommended 30 ml/kg of crystalloid over 3 hours [34], indicating the potential for POCUS use to decrease fluid administration. A randomized controlled trial showed significantly decreased fluid administration in the POCUS group with septic shock as follows: 36 ml/kg in the POCUS group compared to 48 ml/kg in the non-POCUS group [14]. Similar to another systematic review, decreased IVFs in POCUS recipients did yield to significant differences in 28-day mortality, duration of mechanical ventilation, or length of ICU stay between the two groups [35].

The effects of POCUS on the volume status and fluids given do not often appear to be quantified explicitly in the literature [36, 37], but the connection is often implied through clinical intuition. Conceptually, studies that have shown improved clinical outcomes in septic patients who received POCUS theorize the benefits to be largely related to lessened fluid overload [9], a proxy for fluid administration. While not numerous, other studies addressing the effect of POCUS have tended to demonstrate decreased amounts of fluid given to septic patients who received POCUS evaluations, different from our findings. As our POCUS cohort selected for patients with greater disease burden, it is possible that patients with more severe physiological derangements will receive more cumulative fluid and confound the true effects of POCUS examination. The reasons are unknown but may be due to prolonged resuscitation or clinical inertia to intervene more intensely [38, 39]. Unfortunately, the timeline of fluid administration is not known, making it difficult to identify whether POCUS findings altered fluids given or clinical decision making. Furthermore, work controlling for variables of illness severity in the form of randomized controlled trials should be developed to clarify the utility and effect of POCUS in fluid management.

Finally, our data suggest no difference in mortality or oxygen requirements between the POCUS and non-POCUS cohorts. The effects of POCUS on patient mortality seem to be unclear, with some studies suggesting diagnostic improvement with POCUS while others have failed to identify a significant mortality benefit. Literature suggests that sonographic findings of fluid overload such as B-lines were predictive of hypoxemia and respiratory failure [16, 18, 40]. We were unable to fully consider this in our population because lung ultrasound was not performed on all patients in the POCUS group, which may affect the impact of lung ultrasound independent of cardiac POCUS. Our subgroup analysis suggests that even among patients with CHF and severe renal disease, outcomes including hypoxia and mortality were similar between the POCUS and non-POCUS cohorts. Notably, a systematic review by Yuan et al. reported a reduction in 7-day mortality (15% versus 35%, *p*=0.039) in patients who received POCUS-guided fluid resuscitation versus standard of care [35], yet found no statistically significant difference in the length of intubation, length of ICU stay, or 28-day mortality like other articles [14, 41, 42]. The exact relationship among POCUS, mortality, and oxygen requirement may be especially difficult to tease out in our study, as the timing and impact on medical decision making of POCUS is unknown.

We found similar results in our subgroup analysis of CHF and patients with severe kidney disease. Literature suggests these patient populations are perceived as being “at risk” for fluid overload and as a result are under-resuscitated during sepsis [43]. Our findings support this, as on average patients with CHF and severe renal disease received less IVF in both the POCUS and non-POCUS cohorts. Several retrospective studies suggest that while overall mortality is high, outcomes including intubation and mortality are not significantly different in these patient populations when given similar volumes of fluid (>30 mL/kg) [44–46]. This may be due to the altered hemodynamics in septic shock or the overall high mortality of the disease. The literature directly analyzing the impact of POCUS on these specific patient populations in the setting of severe sepsis is sparse and also requires further investigation.

### 4.1. Limitations

Our study has several limitations. First, the sample size, although powered, is limited to a single academic institution. Our results must be confirmed in a larger, multicenter cohort. Second, only patients with a documented POCUS report were included. The POCUS images were not reviewed by consensus/experts and we assume that the POCUS findings are accurate as reported. In addition, we do not know when along the ED timeline any labs, measurements, or POCUS was performed, which diminishes our ability to draw conclusions regarding the effect (if any) that physiological measurements (e.g., blood pressure) and/or POCUS findings had in ED provider decision making. Third, we do not know whether the clinicians were aware of patients' history of severe renal disease or CHF prior to initiation of fluids and if that knowledge impacted fluid management decisions. Fourth, we selected patients with the most severe forms of sepsis, as defined by the high lactate and hypotension, which may produce bias in the total fluid volume administered. Lastly, our institution has no recognized guidelines for protocolizing POCUS findings in the resuscitation of septic patients. Since the study design was retrospective and noninterventional, we intended to evaluate association only and not a causal relationship. Whether POCUS and history of CHF or severe renal disease affects total amount of fluid administered during septic shock needs further investigation.

## 5. Conclusion

Among ED patients with septic shock, POCUS was more likely to be used in patients with higher lactate, lower blood pressure, and vasopressor use and in those requiring mechanical ventilation. There were no differences in total IVF received, new oxygen requirement, or mortality in the POCUS group compared to the non-POCUS group. There is an urgent need for studies that incorporate POCUS findings with specific resuscitation protocols or guidelines to evaluate the impact of POCUS-informed management in septic shock patients.

## Figures and Tables

**Table 1:** Patient characteristics.

Baseline characteristics Total (*n* = 304)	No POCUS (*n* = 226)	POCUS (*n* = 78)	*p* value
Age [year], median (IQR)	65 (54–76)	65 (56–71)	0.74^1^
Male, *n* (%)	125 (55)	45 (58)	0.72^2^
Race, *n* (%)			
Caucasian	101 (45)	35 (45)	0.38^3^
African American	125 (55)	45 (58)	
Comorbidities, *n* (%)			
CHF	38 (17)	20 (26)	0.09
HTN	137 (61)	49 (63)	0.73
Diabetes	93 (41)	27 (35)	0.31
COPD	24 (11)	12 (15)	0.26
Renal failure (≥stage 3)	27 (12)	13 (17)	0.29
CAD	42 (19)	17 (22)	0.54
Weight (kg), median (IQR)	73.4 (59.0–90.7)	71.8 (62.1–84.8)	0.86^1^
Highest lactate, median (IQR)	5.6 (4.7–7.4)	6.7 (5.2–8.7)	0.002^1^
Lowest documented SBP in ED, median (IQR)	85 (73–95)	77.5 (61–86)	<.001^1^
Lowest documented DBP in ED, mean (SD)	48.22 (13.53)	44.51 (13.06)	0.036^*∗*^
Lowest recorded SpO_2_ in ED, median (IQR)	92 (89–94)	92 (87–95)	0.879^1^

CHF, congestive heart failure; HTN, hypertension; COPD, chronic obstructive pulmonary disease; CAD, coronary artery disease; SBP, systolic blood pressure; DBP, diastolic blood pressure; ED, emergency department; SpO_2_, oxygen saturation. Continuous variables are reported as the mean (standard deviation) and median (interquartile range). ^1^*p* value calculated by the Wilcoxon test. ^2^*p* value calculated by the chi-square test. ^3^*p* value calculated by Fisher's exact test. ^*∗*^*p* value calculated by the *T*-test.

**Table 2:** Clinical outcomes occurring during index hospitalization.

Baseline characteristics Total (*n* = 304)	No POCUS (*n* = 226)	POCUS (*n* = 78)	*p* value
Total volume of IVF (mL) during entire ED duration, median (IQR)	2250 (2000–3000)	2500 (1100–3500)	0.781^1^
Total volume IVF in ED (mL/kg), median (IQR)	32.1 (20.15–46.51)	33.02 (16.18–48.37)	0.118^3^
New oxygen requirement during ED stay	133/226 (58.85%)	53/78 (67.95%)	0.155^2^
Positive pressure ventilation at any point in ED	55/226 (24%)	30/78 (38%)	0.017
Vasopressors initiated in ED	76/226 (34%)	40/78 (51%)	0.006^2^
ED disposition, *n* (%)			
Floor	58 (25.7)	12 (15.4)	0.156^3^
ICU	160 (70.8)	64 (82.1)	
Deceased	8 (3.5)	2 (2.6)	
Hospital disposition, *n* (%)			
Discharge	165 (73.0)	53 (68.8)	0.48^2^
Deceased	61 (27.0)	24 (31.2)	

IVF, intravenous fluid; ED, emergency department; ICU, intensive care unit. Continuous variables are reported as the mean (standard deviation) and median (interquartile range). ^1^*p* value calculated by the Wilcoxon test. ^2^*p* value calculated by the chi-square test. ^3^*p* value calculated by Fisher's exact test.

**Table 3:** Subgroup analysis.

Total (*n* = 304)	No POCUS	POCUS	*p* value
CHF patients only	*n* = 38	*n* = 20	
Total volume of IVF (mL) during the entire ED duration, median (IQR)	2000 (1000–3000)	1550 (750–3000)	0.505^1^
Total volume IVF in ED (mL/kg), median (IQR)	20.17 (10.76–42.37)	24.34 (8.29–36.51)	>0.999^1^
Highest lactate, median (IQR)	5.75 (4.5–8.4)	6.2 (4.85–7.6)	0.629^1^
Lowest documented SBP in ED, median (IQR)	81.5 (66–87)	73 (56–78)	0.033^1^
Lowest documented DBP in ED, mean (SD)	45.05 (12.44)	38.2 (10.11)	0.038^*∗*^
Lowest recorded SpO_2_ in ED, median (IQR)	91 (87–95)	90.5 (83.5–94)	0.301^1^
Vasopressors initiated in ED	18/38 (47.37%)	11/20 (55.0%)	0.581^2^
Renal failure (>stage 3) or on hemodialysis patients only	*n* = 27	*n* = 13	
Total volume of IVF (mL) during the entire ED duration, median (IQR)	1500 (1000–2755)	1500 (1000–3056)	0.506^1^
Total volume IVF in ED (mL/kg), median (IQR)	17.54 (7.06–36.9)	23.81 (8.08–37.93)	0.583^1^
Highest lactate, median (IQR)	6.1 (4.7–8.2)	6 (4.9–7.4)	0.896^1^
Lowest documented SBP in ED, median (IQR)	84 (73–95)	75 (61–85)	0.115^1^
Lowest documented DBP in ED, mean (SD)	47.26 (12.09)	46 (10.05)	0.747^*∗*^
Lowest recorded SpO_2_ in ED, median (IQR)	91 (86–94)	95 (92–95)	0.165^1^
Vasopressors initiated in ED	13/27 (48.15%)	8/13 (61.54%)	0.427^2^

IVF, intravenous fluid; ED, emergency department; ICU, intensive care unit. Continuous variables are reported as the mean (standard deviation) and median (interquartile range). ^1^*p* value calculated by the Wilcoxon test. ^2^*p* value calculated by the chi-square test. ^*∗*^*p* value calculated by the *T*-test.

**Table 4:** Logistic regression for predictors of POCUS in the emergency department.

Univariable analysis Total (*n* = 304)	OR (95% CI)	*p* value
Comorbidities, *n* (%)		
CHF	1.71 (0.92–3.16)	0.09
HTN	1.10 (0.64–1.87)	0.73
Diabetes	0.76 (0.44–1.29)	0.31
COPD	1.53 (0.72–3.23)	0.26
Renal failure (>stage 3) or HD	1.47 (0.72–3.02)	0.29
CAD	1.22 (0.65–2.3)	0.54
Total volume IVF in ED (mL/kg)	1.00 (0.99–1.02)	0.47
Highest lactate	1.11 (1.04–1.20)	0.003
Lowest documented SBP in ED	0.98 (0.96–0.99)	0.001
Lowest documented DBP in ED	0.98 (0.96–1.00)	0.038
Lowest recorded SpO_2_ in ED	0.98 (0.96–1.01)	0.20
New oxygen requirement during ED stay	1.48 (0.86–2.55)	0.16
Positive pressure ventilation at any point in ED	1.94 (1.12–3.36)	0.018
Vasopressors initiated in ED	2.08 (1.23–3.50)	0.006
ED disposition, *n* (%)		
Floor	Reference	0.15
ICU	1.93 (0.97–3.84)	
Deceased	1.21 (0.23–6.42)	
Hospital disposition, *n* (%)		
Discharge	Reference	0.48
Deceased	1.22 (0.70–2.15)	

CHF, congestive heart failure; HTN, hypertension; COPD, chronic obstructive pulmonary disease; CAD, coronary artery disease; SBP, systolic blood pressure; DBP, diastolic blood pressure; SpO_2_, oxygen saturation; IVF, intravenous fluid; ED, emergency department; ICU, intensive care unit.

**Table 5:** Adjusted backward logistic regression for predictors of POCUS in the emergency department (only final model covariates shown).

Variable	OR (95% CI)	*p* value
Highest lactate	1.11 (1.03–1.21)	0.008
Lowest documented SBP in ED	0.98 (0.97–0.99)	0.008

SBP, systolic blood pressure; ED, emergency department.

## Data Availability

The data used to support the findings of this study are available from the corresponding author upon request.

## Supplementary Materials

Supplementary Materials Supplemental File 1: STROBE Statement—Checklist of items that should be included in reports of cohort studies.
